# Role of TLR4 activation and signaling in bone remodeling, and afferent sprouting in serum transfer arthritis

**DOI:** 10.1186/s13075-024-03424-4

**Published:** 2024-12-18

**Authors:** Gilson Goncalves dos Santos, Juan Miguel Jiménez-Andrade, Enriqueta Muñoz-Islas, Mariana E. Candanedo-Quiroz, Andrea Gonzalez Cardenas, Bronwen Drummond, Peter Pham, Gwendalynn Stilson, Chao-Chin Hsu, Lauriane Delay, Juliana M. Navia-Pelaez, Julia Paes Lemes, Yury I. Miller, Tony L. Yaksh, Maripat Corr

**Affiliations:** 1 Department of Anesthesiology and Pharmacology, University of California, La Jolla, San Diego, CA USA; 2 Unidad Académica Multidisciplinaria Reynosa-Aztlán, UAT, Reynosa, Tamaulipas México; 3 Department of Medicine, University of California, 9500 Gilman Dr. MC 0663, La Jolla, San Diego, CA USA

**Keywords:** Pain, Arthritis, TLR4, Microglia, Inflammation, CGRP

## Abstract

**Background:**

In the murine K/BxN serum transfer rheumatoid arthritis (RA) model, tactile allodynia persists after resolution of inflammation in male and partially in female wild type (WT) mice, which is absent in Toll-like receptor (TLR)4 deficient animals. We assessed the role of TLR4 on allodynia, bone remodeling and afferent sprouting in this model of arthritis.

**Methods:**

K/BxN sera were injected into male and female mice with conditional or stable TLR4 deletion and controls. Paw swelling was scored and allodynia assessed by von Frey filaments. At day 28, synovial neural fibers were visualized with confocal microscopy and bone density assayed with microCT. Microglial activity and TLR4 dimerization in spinal cords were examined by immunofluorescence and flow cytometry.

**Results:**

In the synovium, K/BxN injected WT male and female mice showed robust increases in calcitonin gene related-peptide (CGRP^+^), tyrosine hydroxylase (TH)^+^ and GAP43^+^ nerve fibers. Trabecular bone density by microCT was significantly decreased in K/BxN WT female but not in WT male mice. The number of osteoclasts increased in both sexes of WT mice, but not in *Tlr4*^*-/-*^ K/BxN mice. We used conditional strains with Cre drivers for monocytes/osteoclasts (lysozyme M), microglia (Tmem119 and Cx3CR1), astrocytes (GFAP) and sensory neurons (advillin) for Tlr4^f/f^ disruption. All strains developed similar arthritis scores after K/BxN serum injection with the exception being the *Tlr4*^*Tmem119*^ mice which showed a reduction. Both sexes of *Tlr4*^*Lyz2*^, *Tlr4*^*Tmem119*^ and *Tlr4*^*Cx3cr1*^ mice displayed a partial reversal of the chronic pain phenotype but not in *Tlr4*^*Avi*l^, and *Tlr4*^*Gfap*^ mice. WT K/BxN male mice showed increases in spinal Iba1, but not GFAP, compared to *Tlr4*^*-/-*^ male mice. To determine whether spinal TLR4 was indeed activated in the K/BxN mice, flow cytometry of lumbar spinal cords of WT K/BxN male mice was performed and revealed that TLR4 in microglia cells (CD11b^+^ /TMEM119^+^) demonstrated dimerization (e.g. activation) and a characteristic increase in lipid rafts.

**Conclusion:**

These results demonstrated a complex chronic allodynia phenotype associated with TLR4 in microglia and monocytic cell lineages, and a parallel spinal TLR4 activation. However, TLR4 is dispensable for the development of peripheral nerve sprouting in this model.

**Supplementary Information:**

The online version contains supplementary material available at 10.1186/s13075-024-03424-4.

## Background

Chronic systemic inflammation in rheumatoid arthritis (RA) is characterized by symmetric swelling of both small and large joints, resulting in loss of function, disability, and chronic pain [1, 2] which adversely affect quality of life, restrict movement, and contribute to depression [3, 4]. In RA, inflammatory cytokines and mediators activate osteoclasts which generate periarticular bone erosions and generalized osteoporosis leading to irreversible bone damage and deformities [5, 6]. Although current therapeutics reduce peripheral joint swelling and attenuate bone damage, RA patients may continue to report joint pain [7–9]. While RA pathology does lead early to an inflammatory milieu and joint afferent sensitization, the pain phenotype and its pharmacology goes beyond the classic cascades responsive to the regulation of inflammation [10, 11]. Our preclinical work on joint inflammation and behavior has focused on the K/BxN serum transfer model of arthritis [12]. In this model we found an immediate mechanical allodynia that precedes visible joint swelling and continues unabated in males following resolution of clinical signs with a partial recovery in females. These properties, along with the loss of an anti-inflammatory pharmacology during the post inflammatory pain phase are considered to be characteristic of the hyperpathic states generated by nerve injury pathologies [13, 14].

Subsequently, our focus on neuraxial TLR4 function led us to observe that *Tlr4*^*−/−*^ K/BxN male and female mice demonstrated clinical signs identical to WT K/BxN animals, but resulted in a reliable resolution of the post inflammation pain state in both sexes [15, 16]. This reversal is recapitulated following intrathecal TLR4 antagonism during the acute inflammatory phase [15, 16]. In transgenic K/BxN mice we identified peripheral sprouting of synovial afferents accompanying bone remodeling and osteoclast activation [14]. Accordingly, given the behavioral profile and pharmacology, we posited that neuraxial TLR4 governs transition from an acute inflammatory pain state to a polyneuropathic pain phenotype in both sexes. In the present work, we specifically examined the effects of TLR4 mutation on glial activation, sprouting, bone remodeling and osteoclast activation in the K/BxN serum transfer model. In models of sterile inflammation, like chemotherapy induced neuropathy (CIPN), TLR4 expressed on lipid rafts in spinal microglia have been described as critical for maintaining a chronic pain phenotype [17–19]. Hence, we assessed pain behavior, as well as TLR4 dimerization and lipid raft properties as a measure of activation of spinal microglial cells in WT K/BxN mice.

## Methods

### Mice

Male wild type (WT) C57BL/6 mice were purchased from Harlan (Indianapolis, IN), and were given at least 48 h to acclimate to the vivarium before use. *Tlr4*^*−/−*^ mice were a gift from Dr. Akira (Osaka University, Osaka, Japan)[20]. *Tlr4*^*f/f*^ (Tlr4^tm1.1Karp/J^, stock #024872), and mice with promoter specific driven Cre recombinase expression: Tg(Avil-icre/ERT2)^AJwo/J^, stock #032027; Tmem119em1(cre/ERT2)^Gfng/J^, stock #031820; Lyz2^tm1(cre)Ifo/J^, stock #00478; Tg(Gfap-cre)77^.6Mvs/2 J^, stock #024098; Cx3cr1^tm2.1(cre/ERT2)Jung/J^; stock #020940 mice were obtained from The Jackson Laboratories (Bar Harbor, ME). Mice with Cre constructs were maintained as heterozygous cre and homozygous *Tlr4*^*fl/fl*^. Mice with estrogen inducible cre were treated with 200 µl tamoxifen (Sigma-Aldrich) 10 mg/ml in corn oil injected intraperitoneally for 5 consecutive days one week prior to an experiment. KRN T cell receptor transgenic mice were a gift from Drs. D. Mathis and C. Benoist (Harvard Medical School, Boston, MA, and Institut de Génétique et de Biologie Moléculaire et Cellulaire, Strasbourg, France)[21]. These mice were maintained on a C57Bl/6 background (K/B). Arthritic mice were obtained by crossing K/B with NOD/Lt (The Jackson Lab; stock 001976) to generate K/BxN.

All animal experiments were conducted according to protocols approved by the Institutional Animal Care and Use Committee of the University of California, San Diego and mice were bred/maintained under standard conditions at the University of California, San Diego Animal Facility that is accredited by the American Association for Accreditation of Laboratory Animal Care. Mice were housed up to 4 per standard cage at room temperature and maintained on a 12:12 h light: dark cycle. All behavioral testing was performed during the light cycle. Both food and water were available ad libitum.

### Passive serum transfer of arthritis

Groups of adult K/BxN transgenic mice were bled, the sera pooled, and transferred to recipient mice by intraperitoneal (IP) injection (100 μl on days 0 and 2). As an indicator of inflammation, ankle width was serially measured with a caliper on days 0–6, 9, 12, 15, 18, 21, 24, and 28. On the day of sacrifice the mice were bled and the hind paws were removed and fixed in 10% formaldehyde. The hind paws were trimmed, decalcified, embedded, sectioned and IHC was performed on the ankle joints.

### von Frey behavioral testing

Mechanical withdrawal thresholds were assessed on days 0–6, 9, 12, 15, 18, 21, 24, and 28 using the up-down method [22]. Briefly, animals were placed in clear, plastic, wire mesh-bottomed cages for 45-min prior to the initiation of testing [16]. Tactile thresholds were measured with a series of von Frey filaments (Seemes Weinstein von Frey anesthesiometer; Stoelting Co., Wood Dale, IL, USA) ranging from 2.44–4.31 (0.02–2.00 g). The 50% probability of withdrawal threshold was calculated. Mechanical values for the left and right hind paws were measured and averaged to produce a single data point per day of measurement. Time course curves plotting thresholds vs time were calculated for each animal and presented as the mean ± SEM for each group. For statistical analysis, the area under the curve (AUC) was calculated for each animal where the response thresholds are converted to the percent change from baseline (baseline threshold − treatment threshold)/(baseline threshold) × 100) and the area under the curve between day 0 and the last test day (e.g., day 28) is calculated as the hyperalgesic index and plotted as group mean ± SEM using GraphPad Prism (version 10; GraphPad Software, San Diego, CA, USA) as previously described [23].

### Arthritis scoring

The development of joint inflammation in the paws was evaluated by visual inspection and rated on a scale of 1 to 28 where one point was given for each swollen digit and two points for each swollen ankle or wrist [14]. Time course curves plotting clinical scores vs time were calculated for each animal and presented as the mean ± SEM for each group. For statistical analysis, the area under the curve (AUC) over 28 days was calculated for each animal and presented as the group mean ± SEM.

### Immunohistochemistry of the spinal cord

At sixteen weeks, mice were deeply anesthetized with Beuthanasia-D and perfused intracardially with 0.9% saline followed by 4% paraformaldehyde. The spinal cords and lumbar DRGs were removed, post-fixed, and cryoprotected in 30% sucrose. Lumbar sections (L4–L5) of the spinal cord were cut on a microtome (30 μm) as free-floating sections. Tissue sections were incubated with anti-Iba1 (Ionized calcium binding adaptor molecule 1) antibody (1:1000 Wako, catalog number 01919741) and anti-GFAP (glial fibrillary acidic protein) antibody (1:1000 Chemicon, catalog number MAB360), washed, and then incubated with secondary antibodies conjugated with fluoro-Alexa-488 and Alexa-594 (1:500, Molecular Probes, Eugene, OR). Images were captured by inverted Leica SP5 confocal microscope and quantified by a blinded investigator using ImageJ (National Institutes of Health). GFAP and Iba1 labeling was quantified by measuring the fluorescence intensity converted to optical density (OD) in arbitrary units in laminae I—III of the dorsal horn of the spinal cord in ImageJ. Initially each picture was converted to 8-bit gray scale. We then calibrated each section using the “uncalibrated OD” function. Eight circular regions of interest (diameter = 88 µM) were placed in laminae I—III. The OD from an equivalent area in a white matter of the same slice was subtracted as the background from the average reading.

### Microcomputed tomography

To assess changes in bone parameters, microCT analysis was performed at the trabecular level of the proximal tibia and calcaneus, using a desktop microCT system (Skyscan 1272; Bruker, Belgium). The scanning parameters were: 10 µm voxel size, at 60 kVp and 166 µA with an integration time of 627 ms, according to the guidelines for microCT analysis of rodent bone structure. All the scanner images were reconstructed using NRecon Software (Bruker, Belgium). The trabecular region of interest (ROI) at the distal tibia was evaluated by selecting 2 mm in the vertical axis, after 0.5 mm from the growth plate (reference point). For the calcaneus, analysis ROI was selected using a 0.5 mm^2^ cylinder that was positioned underneath the growth plate of the calcaneus bone. The CT analyzer program (Bruker, Belgium) was used to determine trabecular bone parameters; an automatic segmentation algorithm (CT analyzer) was applied to isolate the trabecular bone from the cortical bone. The parameters used for the trabecular bone were trabecular bone mineral density (BMD), trabecular bone volume rate (BV/TV), and trabecular number (Tb.N). Finally, hydroxyapatite calibration phantoms (250 and 750 mg/cm^3^) were used to calibrate trabecular bone mineral density values (BMD).

### Bone Immunohistochemistry

Once hindpaws were harvested, they were post-fixed for 24 h and placed in 0.01 M PBS (pH = 7.4) at 4 °C until their micro-computed tomography (microCT) analysis was performed. After the microCT analysis was completed, bone samples were placed in a 10% EDTA solution until total decalcification (around two weeks). The degree of decalcification was regularly monitored by plain radiography (Fona X70, Fona, Assago, Italy). Then, decalcified bones were immersed in a 30% sucrose solution at 4 °C for cryoprotection for 48 h. Then, the femora and ankle joints were cut into serial cross-section. (20 μm thick) with a cryostat (Leica CM1900, Leica Biosystems, Il, USA). The ankle joint sections were incubated for 12 h at room temperature with primary antibody against CGRP (1:4000; Sigma Aldrich; catalog number C8198) to label primary afferent sensory peptidergic nerve fibers. Sympathetic nerve fibers were labeled with primary antibody against tyrosine hydroxylase (TH; 1:1000; EMD Millipore; catalog number AB152). Sprouted nerve fibers were labeled with primary antibody against growth-associated protein-43 (GAP43; 1:1,000; EMD Millipore; catalog number AB5220). For activated monocytes/macrophage we use CD68 primary antibody (Macrosialin; 1:3000; Bio Rad; catalog number MCA1957). Subsequently, preparations were washed in PBS and then incubated for 3 h with the secondary antibody Cy3 monoclonal donkey anti-rabbit (1:600; Jackson ImmunoResearch; Catalog number 711–165–152) or Cy2 monoclonal donkey anti-rat(1:400; Jackson ImmunoResearch; Catalog number 712–225–150). Later, tissue sections were washed in PBS, dehydrated through an alcohol gradient (70, 80, 90, and 100%), cleared in xylene, and cover slipped with DPX mounting medium. Nuclear stain 4′,6-diamidino-2-phenylindole (DAPI; 1:20,000; Invitrogen; catalog number D21490) was used to visualize all cell nuclei.

### Quantification of density of nerve fibers in the ankle joint

For quantification of nerve fiber density, approximately 15 separate 20-μm-thick frozen sections were obtained from the ankle joint of each mouse. For each given marker, three sections from one ankle joint were initially scanned at low magnification (× 10) to identify the areas with the highest density of nerve fibers for each marker through an epifluorescence microscope (Axio Scope.A1, Carl Zeiss, Jena, Germany). One image per section was acquired within the medial synovial area (Supplemental Fig. 1). Then, an image was obtained for each section in this area (separated by at least 100 μm) at 40 × magnification with an epifluorescence microscope. The images were analyzed using ImageJ software (National Institutes of Health) and nerve fibers were manually traced by a blinded investigator, using the freehand line tool, to determine the total length of nerve fibers. Data from at least three sections per ankle joint were recorded and averaged. Total volume was calculated by tracing the area of the image and multiplying this area by the thickness of the Sect. (20 μm). Data are expressed as the total length of nerve fibers per volume of the synovium (mm/mm^3^). Representative images from WT and transgenic mice for each marker were captured with a Carl Zeiss scanning confocal laser microscope (Model LSM 800, Jena Germany). Confocal images used for illustration were assembled and labeled using Adobe Illustrator software.

**Fig. 1:**
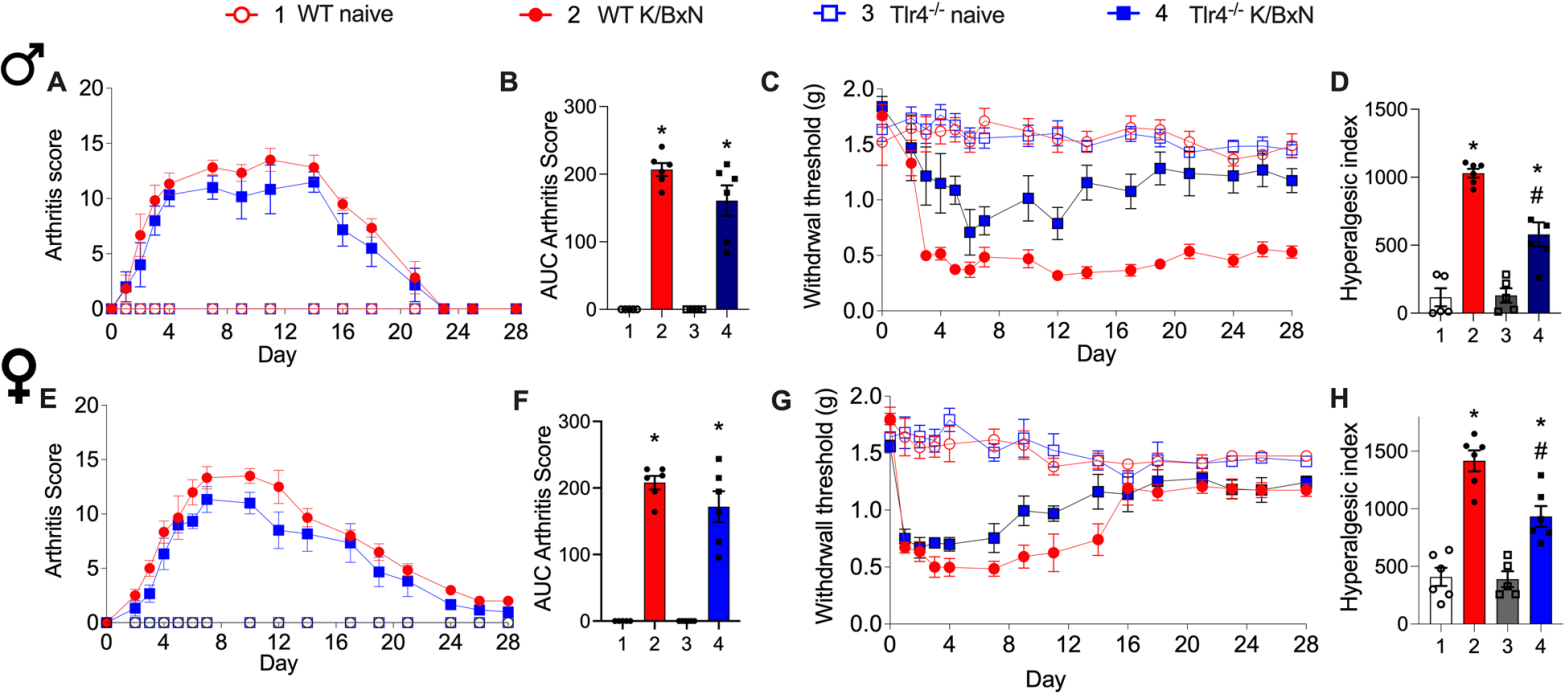
*Differential effects of TLR4 on clinical score and allodynia with K/BxN serum transfer.* Male (**A**-**D**) and female (**E**–**H**) WT mice and *Tlr4*^*−/−*^ mice (were injected on days 0 and 2 with K/BxN sera and developed transiently increased clinical scores with paw inflammation (**A**, **E**) and allodynia (**C**, **G**). The AUCs of aggregate arthritis scores were calculated for all groups. All strains and sexes increased AUCs of arthritis scores compared to their respective naïve controls (**B** and **F**, *p = 0.0001 and one-way ANOVA with Tukey post hoc test). Associated allodynia resolved in the *Tlr4*^*−/−*^ male mice but not in the WT male mice (**C**). The AUCs of aggregate hyperalgesic indices were calculated for all groups. Increased hyperalgesic indices were observed in male and female WT K/BxN mice compared to WT naïve (**D** and **H,** respectively, *p = 0.001 and ^*^p = 0.0001, one-way ANOVA with Tukey post hoc test) and hyperalgesic indices for *Tlr4*^*−/−*^ K/BxN mice compared to WT K/BxN mice, ^#^*p* = 0.007 and *p* = 0.002). Data are represented as mean ± SEM with *n* = 6 per group

### *Quantification of CD68* + *osteoclasts at the trabecular distal femur*

After IHC staining, three confocal images were obtained at 40 × lens with immersion oil. Areas with greater CD68^+^ expression were identified in the trabecular bone at 0.5 mm from the growth plate in the distal femur. Quantification was performed by visualizing all focal planes of Z-axis counting as positive CD68^+^ osteoclast cells, those surrounding the trabeculae of the bone, and having five or more DAPI-stained nuclei. The number of cells was calculated in 1mm^3^ of volume [24, 25].

### Ex-vivo analysis of lipid raft and TLR4 in spinal cord microglia by flow cytometry

The effects of TLR4 deletion are taken as evidence that there is activation of TLR4 signaling. To confirm this, we assessed the presence of TLR4 dimers as an indicator of TLR4 activation. For these *ex-vivo* assays, spinal cords were harvested by hydroextrusion [26] and put on ice while processing. Single-cell suspensions from lumbar tissue were obtained using a Neural Tissue Dissociation kit (Miltenyi Biotec) according to the manufacturer’s protocol. After dissociation cell suspensions were mixed with debris removal solution (Miltenyi Biotec) according to manufacturer’s instructions. To remove myelin, Myelin Removal Beads II (Miltenyi Biotec) were added to samples and incubated for 15 min at 4 °C, followed by separation with LS column and a MACS Separator (Miltenyi Biotec). Following isolation, cells were fixed and blocked with 2% normal mouse serum containing an anti–CD16/CD32 antibody (BD Bioscience; FcγR blocker) for 30 min on ice, followed by staining with an antibody mix of 1:100 PerCP-Cy5.5–conjugated CD11b antibody (BioLegend; RRID:AB_893232), 1:100 PECy7-conjugated rabbit anti-mouse TMEM119 antibody, 1:100 PE-conjugated anti-TLR4 antibody (MTS510 clone Thermo Fisher Scientific; RRID:AB_2562503), 1:100 APC-conjugated anti-TLR4 antibody (SA15-21 clone; BioLegend; RRID:AB_466263), 1:200 dilution of CTxB-FITC (Thermo Fisher Scientific), and 1:1000 Ghost dye-Red 780 fixable viability dye (Cell Signaling) for 45 min on ice. For compensations, single stained beads were used to compensate the signal overlap between channels and unstained cells together with a fluorescence minus one control were used to delineate gates. Data were analyzed by FlowJo (BD Bioscience; RRID:SCR_008520). From these data, we calculated the abundance of lipid rafts measured by cholera toxin B-subunit (CTxB) staining in CD11b + /TMEM119 + microglial cells and their relative change in the number of TLR4 dimers by calculating the ratio of geometric mean of TLR4 monomers (labelled by the MTS510 clone) to the geometric mean of the total TLR4 (labelled by the SA15-21clone) and zero dimers were arbitrarily assigned to unstimulated or naïve cells [17, 27, 28].

### Statistics

Statistical analysis for area under the curve calculations. were performed using GraphPad Prism (version 8.3.0; GraphPad Software, San Diego, CA, USA). The hyperalgesic index is the area under the curve for the percent change from baseline: 100 × ((threshold-baseline threshold)/(baseline threshold)) for the time course (adapted from [29]). Minimum group sizes were targeted to be *n* = 5 animals per group. Group size was found in previous work to be appropriate for establishing as statistically significant changes in paw diameter, clinical score, and tactile thresholds that were considered to be biologically and behaviorally relevant, as compared to vehicle or baseline controls [30–33]. This group size was also considered to be relevant for demonstrating joint sprouting and arthritic bone remodeling based on previous work [14]. For comparisons, one or two-way ANOVA (repeated measures as required) was used with selected comparisons made using Dunnett’s or Tukey post hoc test as noted. Differences reaching or exceeding *p* < 0.05 were considered significant.

## Results

### TLR4 deficiency prevents chronification of mechanical allodynia and glial activation in the K/BxN serum transfer model

Using the K/BxN serum transfer model we measured mechanical withdrawal thresholds and scored arthritis up to 28 days after inducing arthritis (Fig. 1A-H). We reproduced prior results demonstrating the development of maximal clinical signs (arthritis scores) between days 5 and 20 for male and female wild type (WT) and *Tlr4*^*−*/−^ mice (Fig. 1A, B, E, F). There was no difference for aggregated AUCs for arthritis scores between K/BxN males and females (Fig. 1B, F; *p* = 0.14 and *p* = 0.23, respectively). Measurement of mechanical thresholds showed WT males and females displaying a rapid and persistent fall (mechanical allodynia), with a partial recovery in WT female mice (Fig. 1C, D, G, H). In contrast both male and female *Tlr4*^*−/−*^ mice displayed a significant and persistent recovery (e.g. an absence of pain chronification (Fig. 1C, D, G, H). The AUCs of aggregate hyperalgesic indices indicated decreased AUC for *Tlr4*^*−/−*^ K/BxN mice compared to WT K/BxN mice for both males and females (Fig. 1D, H; *p* = 0.007 and *p* = 0.002, respectively).

We previously demonstrated that WT male, but not female mice had a sustained increase in Iba-1 staining in the lumbar spinal cord at 28 days [16]. At day 28 spinal cords from male WT and *Tlr4*^*−/−*^ serum injected mice were examined for glial activation by immuno-reactivity in laminae I-III for GFAP (astrocytes) and Iba-1 (microglia) (Supplemental Fig. 2A-C). Iba-1 staining was increased in serum injected WT mice compared with WT naïve mice (*p* = 0.01, one-way ANOVA), but not in serum injected *Tlr4*^*−/−*^ mice (*p* > 0.05, one-way ANOVA). In contrast, no increase was found in GFAP staining in WT or *Tlr4*^*−/−*^ mice at 28 days (*p* = 0.97, one-way ANOVA).

**Fig. 2:**
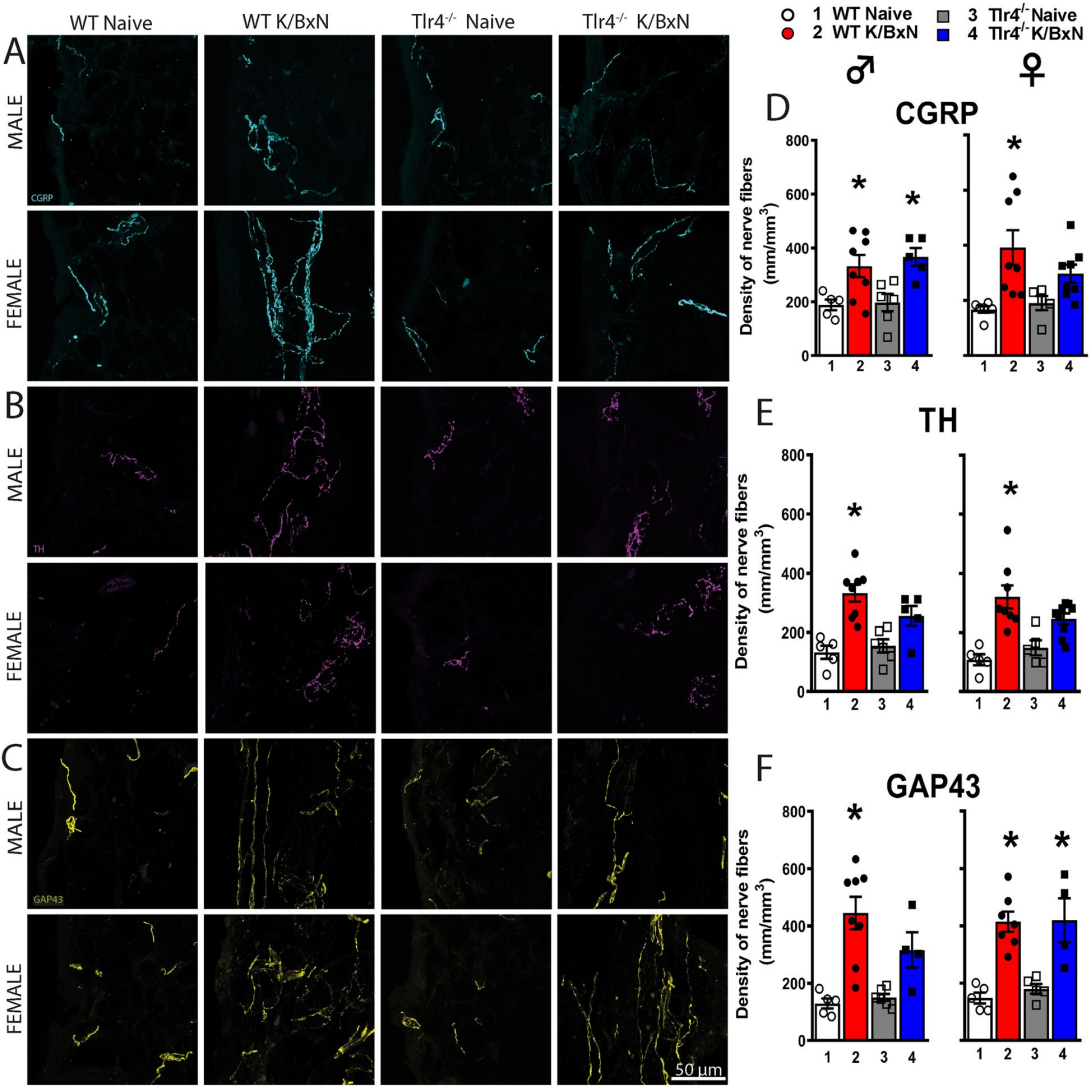
*Arthritic mice have increased density of CGRP* + *, TH* + *and GAP43* + *nerve fibers in the ankle joint*. Representative confocal images of CGRP (**A**, cyan), TH (**B**, purple) and GAP43 (**C**, yellow) in ankle-joint sections (20 μm-thick) from naïve and serum injected WT and *Tlr4*^*−/−*^ male and female mice. Significantly greater densities of CGRP^+^ (**D**), TH^+^ (**E**), and GAP43^+^ (**F**) nerve fibers were quantitated in WT serum injected compared to naïve males (*p* = 0.46; *p* = 0.003; *p* = 0.005 respectively) and females (*p* = 0.007, *p* = 0.0003, *p* = 0.0005 respectively). Male *Tlr4*^*−/−*^ serum injected mice had a significant increase in the density of CGRP^+^ (**D**; *p* = 0.03), but not TH^+^ (**E**; *p* = 0.10) fibers and GAP43^+^ (**F**; *p* = 0.14) fibers, compared to naïve controls. Female *Tlr4*^*−/−*^ serum injected mice had a significant increase in the density of GAP43^+^ (**F**; *p* = 0.0046), but not in CGRP^+^ (*p* = 0.38) or TH.^+^ fibers (*p* = 0.12) compared to naïve controls. *p < 0.05 one-way ANOVA with Tukey post hoc test (*n* = 4–8 per group)

### Both sexes of WT and Tlr4^−/−^ mice display robust increases in nerve *fiber* density in arthritic ankles

The ankle joints of the arthritic and naïve *Tlr4*^*−/−*^ and WT male and female mice were examined by immunohistochemistry for the presence of markers for sympathetic, peptidergic and sprouted nerve fibers at 28 days. In naïve male and female WT mice, a regular pattern of low-level innervation by CGRP^+^, TH^+^ and GAP43^+^ nerve fibers was observed in the synovium (Fig. 2A-F). In contrast, K/BxN serum injected WT mice displayed a significant increase as compared to WT naïve in the density of CGRP^+^ (*p* = 0.047), TH^+^ (*p* = 0.0003), and GAP43^+^ (*p* = 0.0005) nerve fibers. These images show this increase displayed a disorganized appearance as compared with naïve mice (Fig. 2A-C). Injection of K/BxN serum in male *Tlr4*^−/−^ mice also induced a significant increase of CGRP^+^ (*p* = 0.025; Fig. 2A and D) as compared to male *Tlr4*^*−/−*^ naïve mice. Female *Tlr4*^*−/−*^ serum injected mice had a significant increase in the density of GAP43^+^ (*p* = 0.0046; Fig. 2F), but not in the density of CGRP^+^ or TH^+^ fibers (Fig. 2D, E; *p* = 0.38 and *p* = 0.12, respectively). There were no statistical differences between the males and females for each treatment group including the density of CGRP^+^ fibers in between the injected male and female *Tlr4*^*−/−*^ mice (*p* = 0.06, Fig. 2D). These results indicate peripheral nerve sprouting in arthritic joints from WT mice in both sexes that also occurred in *Tlr4*^*−/−*^ mice.

### Recovery of bone loss at day 28 by microCT analysis of arthritic mice

**Fig. 3:**
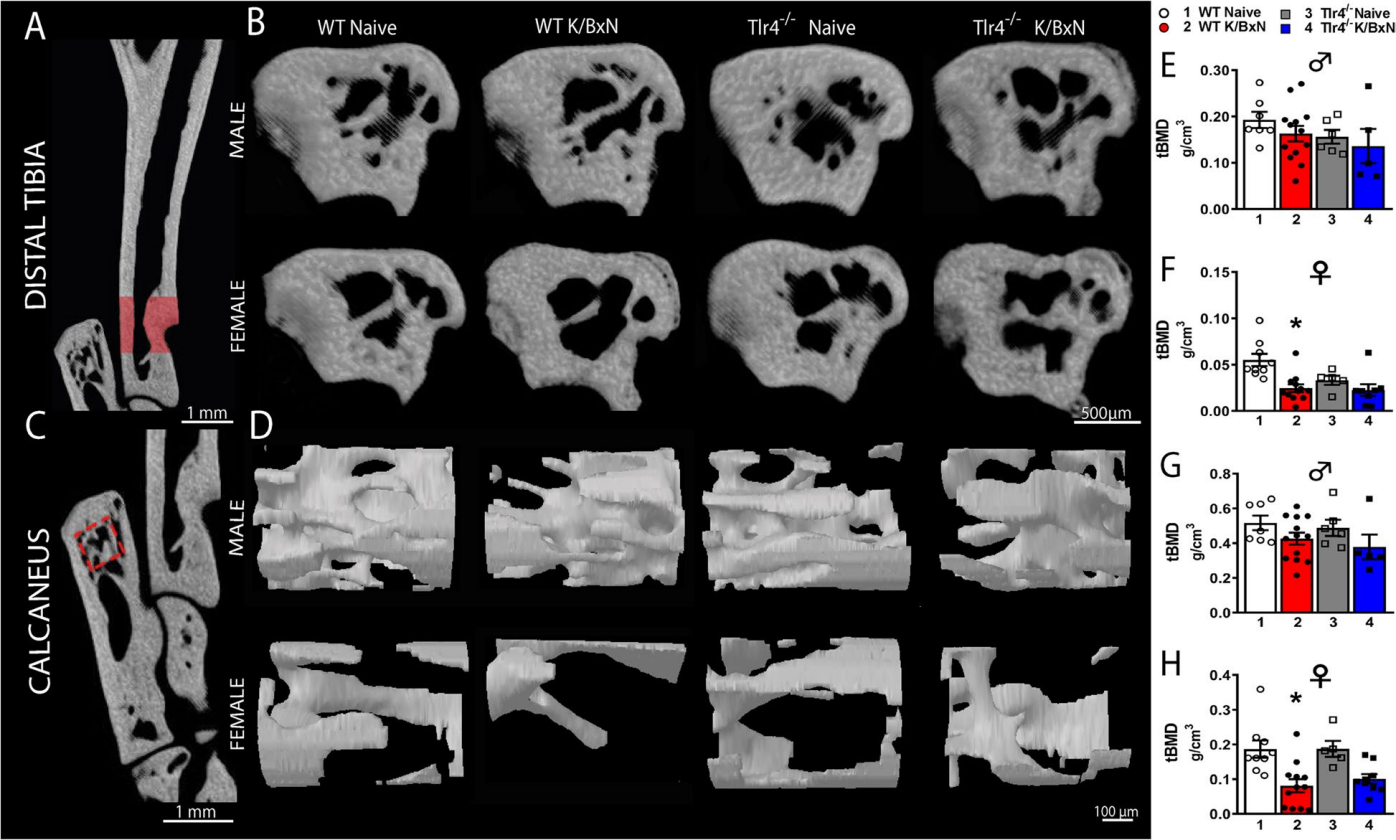
*Differences in trabecular bone parameters from distal tibia and calcaneus in WT and Tlr4*^*−/−*^* arthritis mice.* Representative images of tridimensional reconstructions from trabecular bone parameters of distal tibia (**A**, **B**) and calcaneus (**C**, **D**). MicroCT analysis was performed on the tibia and calcaneus of WT, WT serum injected, *Tlr4*^*−/−*^ and *Tlr4*^*−/−*^ serum injected male and female mice. Serum injection decreased tibial (**F**; *p* = 0.0014) and calcaneal (**H**; *p* = 0.0027) trabecular BMD in female but not in male mice (**E**, **G**) at 28 days compared to their respective controls. There were no significant differences in tBMD in the tibia (**E**, **F**) and calcaneus (**G**, **H**) of *Tlr4*.^*−/−*^ serum injected male (**E**, **G**) or female mice (**F**, **H**) at 28 days compared to naïve controls. *p < 0.003 one-way ANOVA with Tukey post hoc test (*n* = 5–12 per group)

In the acute phase of K/BxN arthritis there is rapid bone erosion and bone loss [34]. However, as inflammation subsides remodeling may occur with an erosion repair [35]. To determine if there were persistent changes in bone density at day 28 in the post inflammatory phase, the hind limbs of WT and *Tlr4*^*−/−*^ serum injected and age matched naïve controls were removed for microCT analysis (Fig. 3A-H). We determined trabecular bone mineral density (tBMD) in the distal tibia (Fig. 3A, B, E, F) and calcaneus (Fig. 3C, D, G, H). In WT male mice K/BxN serum, did not result in significant changes of trabecular BMD (tBMD) at day 28, as compared to their respective controls in the distal tibia and calcaneus (Fig. 3E, G; *p* = 0.99 and *p* = 0.54, respectively). However, K/BxN serum injected female mice had decreased tibial (Fig. 3F; *p* = 0.0014) and calcaneal (Fig. 3H; *p* = 0.0027) tBMD compared to their age matched controls (one-way ANOVA with Tukey post hoc test). There were no significant differences in tBMD in male and female *Tlr4*^*−/−*^ serum injected mice in the tibia (Fig. 3E, F; *p* = 0.99 and *p* = 0.99, respectively) and calcaneus (Fig. 3G, H; *p* = 0.60 and *p* = 0.13, respectively) at 28 days compared to naïve controls.

### Increased CD68^+^ osteoclasts at distal femurs in WT but not Tlr4^*−/−*^ arthritic mice of both sexes

**Fig. 4:**
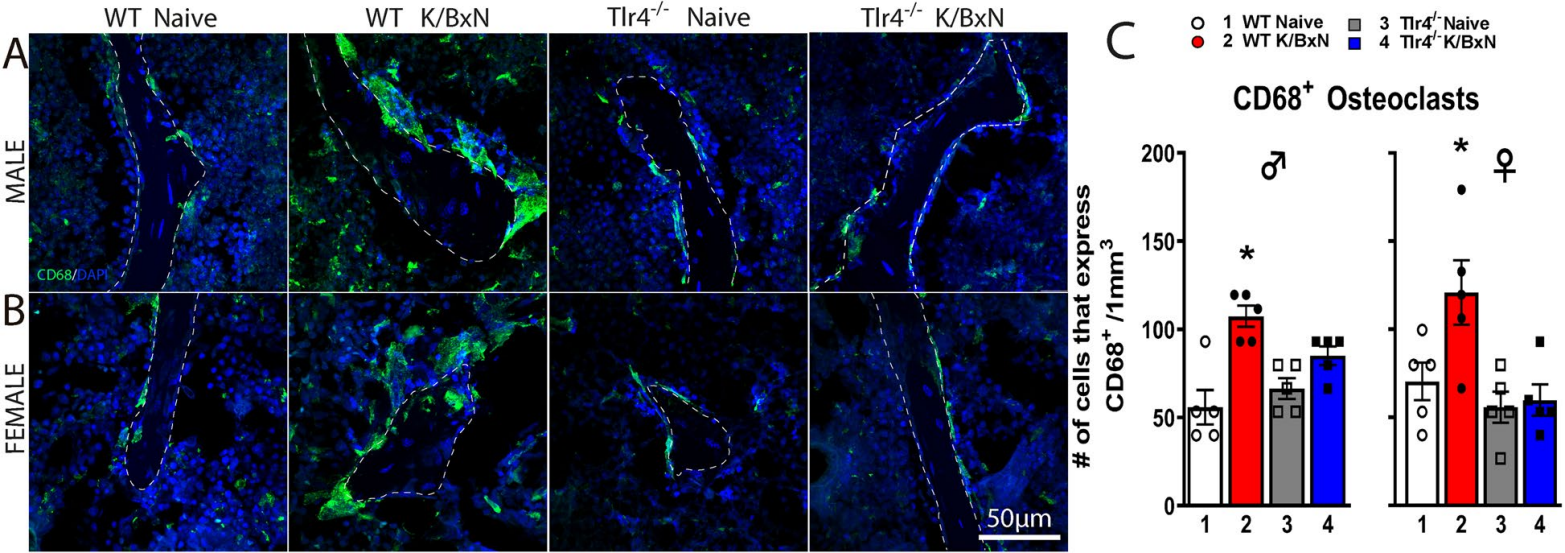
*K/BxN serum injection increases the density of CD68*^+^ *osteoclasts in trabecular distal femurs of WT but not Tlr4*^*−/−*^* mice at 28 days.* Representative confocal images of CD68^+^ cells (green) in male (**A**) and female (**B**) mice of Sects. (20 μm thick) at the distal femoral metaphysis from WT, WT serum-injected, *Tlr4*^*−/−*^ and *Tlr4*^*−/−*^ serum-injected mice. A significantly greater density of multinucleated cells expressing CD68^+^ was found in male (*p* = 0.0004) and female (*p* = 0.04) WT serum injected mice compared with their respective controls (**C**). However, this increase of CD68^+^ osteoclasts was not observed in *Tlr4*^*−/−*^ serum injected male and female mice (**C**). *p < 0.05 one-way ANOVA with Tukey post hoc test; *n* = 5 per group

Osteoclast activity had been previously reported as contributing to the allodynia seen in a murine arthritis [36, 37]. Immunohistochemistry and confocal analyses reveals that K/BxN serum in both WT males and females induced a significant increase in the number of CD68^+^ multinucleated osteoclasts (Fig. 4A-C; *p* = 0.0004 and *p* = 0.04, respectively by one-way ANOVA with Tukey post hoc test). However, this serum-induced increase in osteoclasts was absent in male and female *Tlr4*^−/−^ mice (Fig. 4A-C; *p* = 0.27 and *p* = 0.99, respectively).

### Deletion of TLR4 in lysozyme M expressing cells correlates with reduced pain and inflammation in arthritic mice, despite proliferation of nerve fibers

**Fig. 5:**
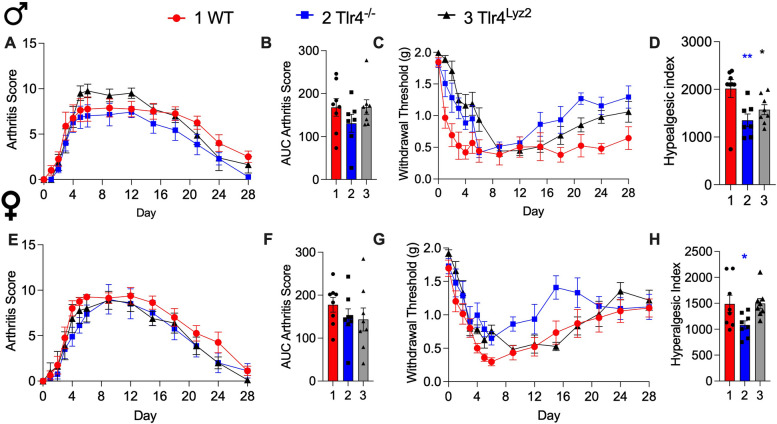
*TLR4 expressed by Lyz2*^+^ *cells modulate pain behavior.* WT, *Tlr4*^*−/−*^ and Tlr4^Lyz2^ mice were injected with K/BxN sera on days 0 and 2 and developed robust arthritis scores (**A**, **E**) without differences between strains (**B**, **F**). WT male mice developed prolonged allodynia whereas *Tlr4*^*Lyz*2^ and *Tlr4*^*−/−*^ mice had reduced mechanical allodynia (F (2, 280) = 39.28; *p* < 0.0001, two-way ANOVA) (**C**) and significantly reduced hyperalgesic indexes (F (2, 20) = 7.51 *p* = 0.004; *p = 0.04 and **p = 0.0022 (**D**). Female WT, *Tlr4*^*Lyz*2^ and *Tlr4*^*−/−*^ mice demonstrated at least partial recovery of the withdrawal thresholds in the late phase (**G**) which was significantly faster in the *Tlr*.^*−/−*^ mice (F (2, 21) = 3.83 *p* = 0.038; *p < 0.04 one-way ANOVA with Dunnett’s post hoc comparison to WT (**H**)

As the WT mice had an increase in CD68^+^ cells in the distal femur that was TLR4 dependent, we sought to determine if myeloid cells, which would include osteoclasts, affected the pain-like behavior. Lysozyme M is expressed by osteoclasts, monocytes and granulocytes [38]. Hence, we examined mice that were heterozygous for Lyz2 (gene for lysozyme M) driven cre recombinase to disrupt *Tlr4*^*f/f*^ (*Tlr4*^*Lyz2*^) in the serum transfer model (Fig. 5 A-H). Despite similar joint scores (Fig. 5A, B); [F (2, 280) = 7.344, *p* = 0.9 two-way ANOVA], the *Tlr4*^*Lyz2*^ male mice displayed a modest, but significantly attenuated allodynia compared to WT mice (Fig. 5C, D; F (2, 280) = 39.28; *p* < 0.0001, two-way ANOVA). AUC indices for withdrawal thresholds indicated that the male *Tlr4*^*−/−*^* mice* and *Tlr4*^*Lyz2*^ mice had similar joint score profiles (Fig. 5B), and reduced allodynia compared to WT male mice (Fig. 5D; (F (2, 20) = 7.51 *p* = 0.004).

There were no differences in the WT, *Tlr4*^*−/−*^ and *Tlr4*^*Lyz2*^ female joint scores (Fig. 5E, F). Female WT, *Tlr4*^*Lyz*2^ and *Tlr4*^*−/−*^ mice demonstrated at least partial recovery of the withdrawal thresholds in the late phase which was significantly faster in the *Tlr4*^*−/−*^ mice (Fig. 5G; (F (2, 21) = 3.83 *p* = 0.038). These results indicate that the TLR4 on lysozyme expressing cells governs the persistence of allodynia in the males but makes a lesser contribution to the recovery rate of females, unlike the mice with a global loss of TLR4.

**Fig. 6:**
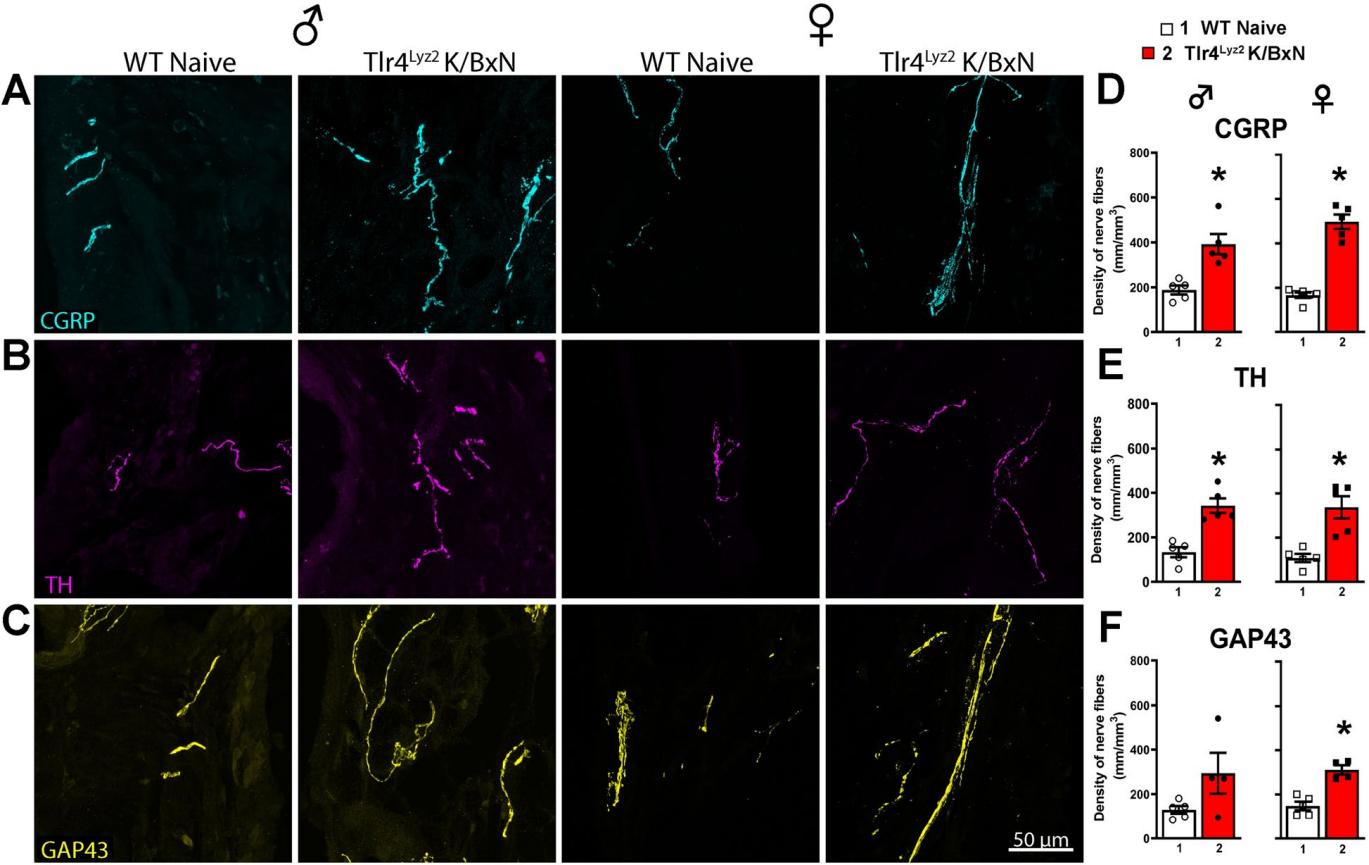
*WT and Tlr4*^*Lyz2*^*male and female serum mice show increases in peripheral nerve fibers.* Representative confocal images of ankle joint Sects. (20 μm-thick) stained for (**A**) CGRP (marker of a subtype of sensory axons; cyan), (**B**) TH (marker of sympathetic axons; purple) and **(C)** GAP43 (marker of nerve fibers undergoing regeneration; yellow). **D**-**F** Male *Tlr4*^*Lyz2*^ mice had a significant increase in TH^+^(*p* = 0.0007) and CGRP^+^ (*p* = 0.0032) fibers but not GAP43^+^ (*p* = 0.088) fibers and female *Tlr4*^*Lyz2*^ mice had significant increases in TH^+^ (*p* = 0.0026), CGRP^+^ (*p* = 0.0001) and GAP43^+^ (*p* = 0.0007) fibers (*p < 0.04, Student t test; *n* = 5–6 per group)

**Fig. 7:**
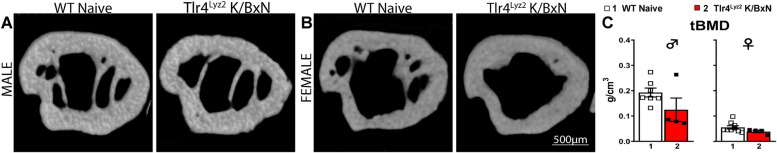
*Differences in trabecular bone parameters from WT and Tlr4 *^*Lyz2*^* arthritis mice.* **A**, **B** Representative images of tridimensional reconstructions from trabecular bone in the distal tibia. **C** No significant change in bone density was detected at day 28 in the *Tlr4*.^*Lyz2*^ male (*p* = 0.13) or female (*p* = 0.15) mice compared to naïve controls (Student t test; *n* = 5–6 per group)

On day 28 the hind limbs were harvested to analyze peripheral innervation (Fig. 6A-F) and bone density (Fig. 7A-C). Male *Tlr4*^*Lyz2*^ mice had a significant increase in TH^+^ (*p* = 0.0007) and CGRP^+^ (*p* = 0.0032) fibers but not GAP43^+^ (*p* = 0.088) fibers (Fig. 6D-F) and the female *Tlr4*^*Lyz2*^ mice had significant increases in TH^+^ (*p* = 0.0026), CGRP^+^ (*p* = 0.0001) and GAP43^+^ (*p* = 0.0007) fibers (Fig. 6D-F) demonstrating peripheral sprouting in this strain. *Tlr4*^*Lyz2*^ mice had no significant changes in trabecular bone density compared with the WT naive mice (Fig. 7; *p* = 0.13 for males and *p* = 0.15 for females). These results support the prior finding that sprouting, and bone density changes are not reliant on TLR4 expressing cells [14].

### Microglial TLR4 modulates pain and inflammation in serum transfer mice

**Fig. 8:**
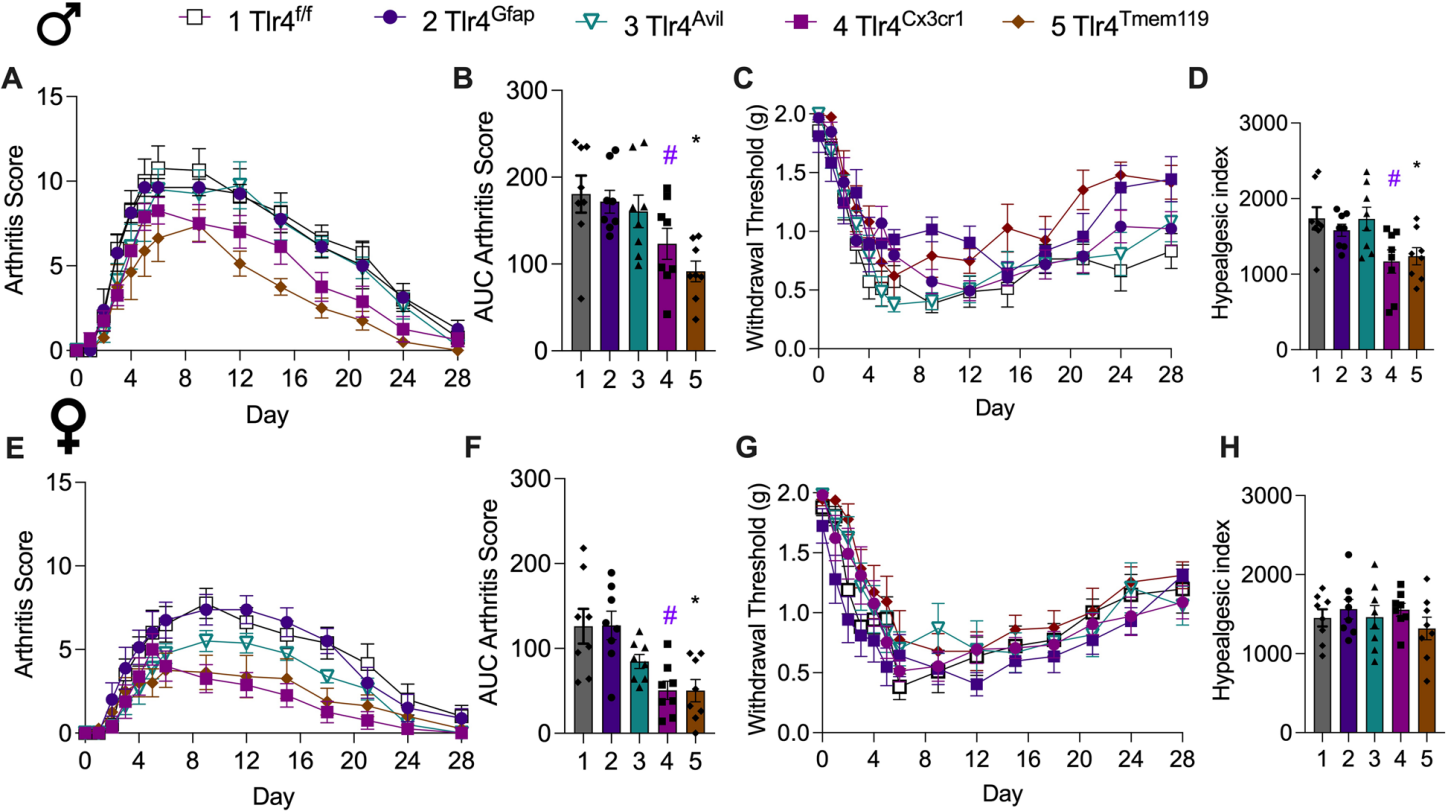
*TLR4 expressed by microglial cells modulates mechanical allodynia.* Male and female *Tlr4*^*Avil*^*, Tlr4*^*Gfap*^*, Tlr4*^*Cx3cr1*^* Tlr4*^*Tmem119*^*, Tlr4*^*f/f*^ mice (*n* = 8/group) were injected on days 0 and 2 with K/BxN sera and serially scored for arthritis (**A**, **B**, **E**, **F**) and assessed for mechanical withdrawal (**C**, **D**, **G**, **H**). (**A**, **B**, **E**, **F**) All mice developed visually detectable paw swelling*.* (B) Overall, there were differences over the time course in males (F (4, 35) = 5.911, *p* < 0.001 one-way ANOVA), notably for *Tlr4*^*Tmem119*^ (*p = 0.039) *and Tlr4*^*Cx3cr1*^ (^#^*p* = 0.0009) compared to *Tlr4*^*f/f*^ controls with Dunnett’s test. (**F**) Similarly for female mice (F(4,35) = 5.91, *p* = 0003) there were significant differences in *Tlr4*^*Tmem119*^ (*p = 0.0028) *and Tlr4*^*Cx3cr1*^ (^#^*p* = 0.0027). (**C**) The withdrawal thresholds for *Tlr4*^*Tmem*^ and *Tlr4*^*Cx3cr1*^ male mice partially returned to baseline with significant differences at the end of the time course (F (4, 490) = 14.94, *p* < 0.0001). (**D**) The hyperalgesic indexes were significantly reduced overall (one-way ANOVA, *p* = 0.0058) for *Tlr4*^*Tmem119*^ (* *p* = 0.03) and *Tlr4*^*Cx3cr1*^ (^#^*p* = 0.02) male mice compared to *Tlr4*.^*f/f*^ controls with Dunnett’s test. (**G**, **H**) There were no differences in the withdrawal thresholds and the hyperalgesic indexes in the female mice (F(4, 35) = 0.65; *p* = 0.63, one-way ANOVA)

In the periphery there was an increase in neural fiber density in both *Tlr4*^*−/−*^ and WT mice, suggesting that other mechanisms were involved in TLR4 influencing the late phase phenotype in WT mice. Although there were no lasting changes to bone density, osteoclasts could be contributors as seen in other antibody mediated arthritis models [36, 37, 39]. However, lysozyme is expressed at differing levels by many other cell types including neutrophils and macrophages in the joint and in the DRG [40, 41]. Accordingly, additional strains with *Tlr4*^f*/f*^ were examined with cre recombinase driven by advillin to target sensory neurons, GFAP for astrocytes and DRG satellite cells, Cx3Cr1 for microglia and DRG macrophages, and TMEM119 for resident microglia (Fig. 8A-H). Among these strains the *Tlr4*^*Avil*^ and *Tlr4*^*Gfap*^ mice did not have significant effects on either clinical signs or mechanical allodynia in the late phase of the serum transfer model in either males or females. However, in males *Tlr4*^*Tmem119*^ (*p* = 0.039) *and Tlr4*^*Cx3cr1*^ (*p* = 0.0009) resulted in a significant reduction in clinical signs (Fig. 8A,B). Similarly in female mice in *Tlr4*^*Tmem119*^ (*p* = 0.0028) *and Tlr4*^*Cx3cr1*^ (*p* = 0.0027) had lower scores (Fig. 8E,F). The withdrawal thresholds for *Tlr4*^*Tmem119*^ and *Tlr4*^*Cx3cr1*^ male mice partially returned to baseline with significant differences at the end of the time course (F (4, 490) = 14.94, *p* < 0.0001) with reduced allodynia seen for *Tlr4*^*Tmem119*^ (*p* = 0.03) and *Tlr4*^*Cx3cr1*^ (*p* = 0.02) compared to *Tlr4*^*f/f*^ controls (Fig. 8C, D) There were no differences in the withdrawal thresholds (Fig. 8G) and the hyperalgesic indexes in the female mice (F(4, 35) = 0.65; p 0.63, one-way ANOVA) (Fig. 8H). These results, while not excluding a contribution by macrophages or other cre expressing cells, implicate TLR4 on microglial cells as playing a role in the late phase phenotype in the male.

### Microglia from arthritic mice show an increased TLR4 dimerization and lipid rafts

**Fig. 9:**
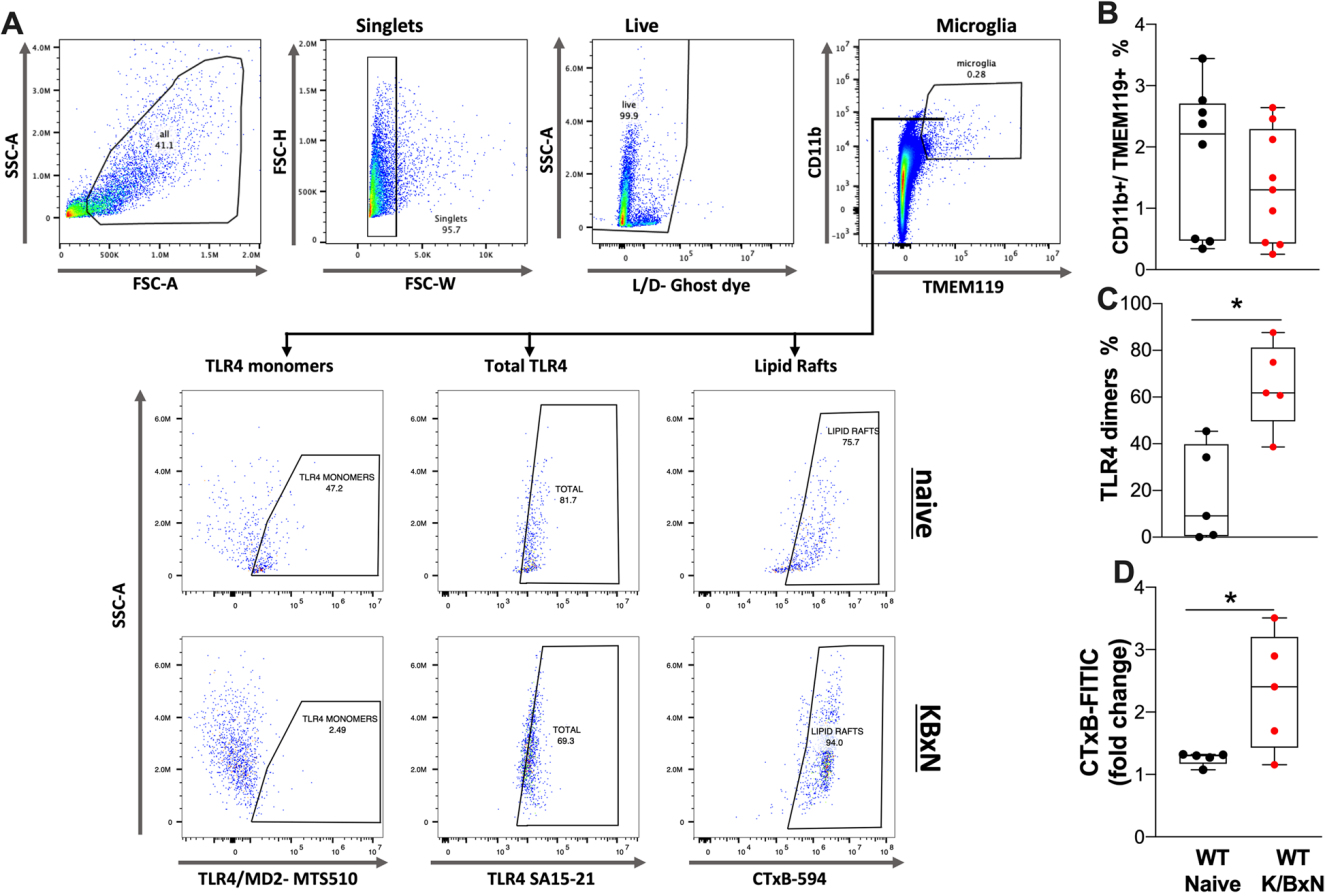
*Microglia from arthritic mice show increased TLR4 dimers and lipid rafts.* Single cell suspensions from spinal cords were generated from naïve and WT K/BxN male mice injected with serum after 28 days and stained for microglia markers, TLR4 and lipid rafts. (**A**) Gating strategy and representative plots of microglia (CD11b + /TMEM119 +) from K/BXN serum injected and naïve mice showing cell population intensity (mean fluorescence intensity), for TLR4 monomers (MTS510 clone), total TLR4 (SA15-21 clone) and lipid rafts (CTxB binding). Quantification of microglia populations (**B**), TLR4 dimers (**C**) and change in lipid rafts (**D**) are shown (*n* = 5–9 per group). There were no significant changes in the numbers of CD11b + /TMEM119 + cells at 28 days; however, the percentage of TLR4 dimers (*p* < 0.015) and increase in the lipid rafts were significant (*p* = 0.033, Students t test)

TLR4 is robustly expressed in microglia [15, 18, 42] and their activation (marked by their dimerization) leads to robust microglial activation [18, 43–46]. As WT mice displayed an increase in spinal cord microglial immunoreactivity, we sought to measure TLR4 activation using flow cytometry in spinal microglia during the post-inflammatory phase of K/BxN arthritis. Activation was assessed by measuring TLR4 dimerization and changes in the lipid rafts in which they are preferentially expressed (Fig. 9A, B). The percentage of resident microglia (CD11b^+^/TMEM119^+^) at day 28 was not significantly different from naïve mice (*p* = 0.65, unpaired Student t‐test). However, the microglial cells from serum injected WT male mice showed an increase in TLR4 dimerization (*p* = 0.015, Student t‐test, Fig. 9C) and lipid raft content (*p* = 0.033, Student t‐test, Fig. 9D). These results suggest that microglial activation as indicated by TLR4 dimerization within the lipid raft may play a pivotal role in regulating the persistence of pain-like behavior in the post inflammatory phase of arthritis.

## Discussion

In the present work, in the K/BxN mouse, we examined at 28 days, ankle joints and trabecular bone for changes including bone density, osteoclast activation, and sprouting of synovial innervation. In spinal dorsal horn we utilized immunohistochemistry and flow cytometry. In these studies, we observed prominent changes in bone density, evidence of synovial sprouting and of particular note, ongoing activation of spinal TLR4 signaling, as evidenced by *persistent* increases in TLR4 dimerization and lipid raft size.

### Role of TLR4 in persistent pain in arthritis

In longstanding inflammatory arthritis, patients experience persistent pain despite clinically inactive disease. There are multiple mechanisms that likely contribute to this clinical scenario including peripheral nerve sprouting distally at the joint and sensitization of the central nervous system. Our prior work indicated that TLR4 played a key role in establishing unremitting allodynia after resolution of acute inflammation in male, but not female mice in the K/BxN serum transfer model [15, 16, 47]. TLR4 deficiency had no effect on baseline thresholds, but significantly attenuated the late phase allodynia seen in both sexes of WT mice resulting in a return to a non-allodynic baseline [15, 16, 47]. Concordantly, inhibition of TLR4 at the peak of inflammation reduced allodynia transiently [15]. Hence, we focused our analyses on mice at 28 days post serum injection for the persistent effects related to TLR4 function.

### Sprouting of synovial innervation

Although peripheral nerve sprouting has been associated with neuropathic conditions, it is still unknown if this proliferation plays a substantial role in RA pain. Based on prior reports using several models of musculoskeletal pain, nerve fiber sprouting has been correlated with neuropathic pain-like behavior [14, 48–52]. In the present work WT naïve mice displayed a regular pattern of organized CGRP^+^ and TH^+^ nerve fibers in the ankle synovium. In arthritic WT male and female mice there was an increase in the density of CGRP^+^, TH^+^, and GAP43^+^ nerve fibers which also had a ramified and disorganized appearance. CGRP is uniformly considered to reflect small afferent innervation and its presence in the periphery and superficial dorsal horn is highly suggestive transmission of nociceptive information [53–55]. However, TH has also been extensively used as marker for sympathetic post-ganglionic innervation to study peripheral sprouting in pathological conditions [14, 56, 57]. Our findings are consistent with previous studies in humans and rodents indicating that remodeling of sensory and sympathetic nerve fibers can be associated with musculoskeletal pain [48, 49, 51, 56–60]. While the number of CGRP^+^ and TH^+^ fibers were numerically increased in female *Tlr4*^*−/−*^ mice, these changes were not statistically significant. The male *Tlr4*^*−/−*^ mice also did not have a significant increase in TH^+^ fibers suggesting that TLR4 might play a role in the peripheral sprouting of sympathetic nerve fibers under inflammatory conditions [53, 61].

Recently a CGRP antagonist, olcegepant, was used to prevent the onset of swelling and subsequent tactile allodynia in the early phase of the K/BxN model with a pretreatment regimen in female mice [31]. As *Tlr4*^*−/−*^ mice reverse the pain phenotype despite the presence of increased CGRP and GAP43 innervation in the synovium, the role of CGRP antagonism in regulating long standing inflammatory arthritis remains to be addressed. It should be emphasized that the current studies focus on the established post-inflammatory phase. Work by Botz and colleagues have pointed to the anti-inflammatory role of joint neuropeptide release mediated by neurogenic antidromic activation in the very early phases of K/BxN inflammation [62].

### Osteoclastogenesis

In addition to neural activity CGRP influences osteogenesis via its action on osteoblasts [63]. However, in the present study, we also observed an increase in the number of osteoclast cells (multinucleated CD68^+^ cells) in the femurs from late phase WT arthritic mice, which was normalized in *Tlr4*^*−/−*^ animals. These findings correspond to a prior report where TLR4 deficiency protected against bone destruction and impaired osteoclast formation with a paucity of TRAP-positive cells seen in *Tlr4*^*−/−*^ mice [64]. Although an increase in the number of CD68^+^ osteoclasts was observed in the WT mice, our microCT analyses demonstrated differences in trabecular bone loss only in the female WT mice at day 28. These findings are consistent with our prior histologic findings in the K/BxN model demonstrating marked bone erosion in the early phase; however, weeks after the inflammation resolves there can be little detectable chronic bony damage in male mice [35]. Others have suggested that the role of osteoclasts is independent of active inflammation and may lie in their ability to produce chemokines [37]. It remains unclear if CGRP plays any role in the resolution of bony changes in this model as CGRP + fibers were present in both males and females, yet only females demonstrated significant bone loss.

### Glial activation

We have shown that an activated state of spinal glial cells after resolution of inflammation contributes to the persistent pain phenotype [14, 16]. Although TLR4 plays a key role in models of mono and polyneuropathy [16, 17, 32, 65] there may be multiple cell types involved. In the current data deletion of *Tlr4* using advillin and GFAP driven cre promoters did not significantly influence the development of swelling or pain-like behavior in either sex. Deletion of *Tlr4* using cre promoters that would affect spinal microglia and other cells from the monocyte lineage suggest that these cells play a dominant role in establishing and sustaining the long-term pain phenotype in male mice. Although there was a reduction in arthritis scores there was no significant change in the time course of withdrawal thresholds in the females. Here we did not examine the activation of DRG macrophages in the late phase, which others have reported as key in the onset and persistence of pain associated with inflammation [40, 41, 66].

### TLR4 and microglia

Recently, we have described the involvement of TLR4 in spinal microglia in CIPN and nerve injury, highlighting this cell population as major player in chronic pain [18]. Here we broaden our results, highlighting that during arthritis development, TLR4 dimerization in microglia within lipid rafts may contribute for arthritic pain processing too [17, 18]. As an added consideration, these observations provide for the first time an indication that even in the late phase there is persistent activation of TLR4 in the neuraxis. This property bears close resemblance to the phenotype observed in other poly neuropathies such as the chemotherapy induced peripheral neuropathy (CIPN) model [18]. Indeed, WT arthritic mice have an increase in spinal cord microglia immunoreactivity (Iba1) which was reduced in the absence of TLR4. Moreover, microglia phenotype was associated with augmented lipid raft content and dimerization suggesting that after resolution of inflammation microglia display an inflammatory phenotype that correlated with the chronic pain phenotype. Indeed, *Tlr4* deletion on microglia (*Tlr4*^*Tmem119*^ and *Tlr4*^*Cxcr1*^ mice) partially reversed the chronic pain phenotype in the late phase in male mice but had minimal if any effect in female mice demonstrating a key role of TLR4 function in microglia with the evolution of a chronic pain phenotype in males in the K/BxN serum transfer mice.

### Study limitations

We note that conditional deletion may not recapitulate the phenotype seen in mice that have a germline disruption in the same gene [67]. Often mice will compensate for the loss of gene expression by a variety of mechanisms that will influence the phenotype in the adult animal [68]. An inducible cre promoter results in the acute loss of gene expression and may not always replicate the phenotype of a global knockout. An additional limitation of the use of cre driven recombination is the expression is not absolutely restricted to single cell types. Also, it should be emphasized that these results do not exclude a role for the facilitatory effects of DRG macrophages on the signaling in pain states and suggest the combined effects of TLR4 signaling on these two cell populations in driving the persistent pain phenotype. These results, however, corroborate our prior findings, where we show that spinal TLR4 modulates TNF production [16] and highlight the pivotal involvement of microglial TLR4 in pain processing that may evolve into a chronic neuropathic pain state.

## Conclusions

The murine K/BxN serum transfer model of arthritis is associated with increased peripheral sprouting in male and female mice and minimal if any post-inflammatory bone loss in male mice. Both sexes of WT mice have a persistent increase in osteoclasts at day 28 unlike TLR4 deficient mice which may contribute to persistent pain behavior independent of active inflammation. In male mice the TLR4 on monocytes and microglia, but not astrocytes, is involved in pain processing and inflammation. TLR4 dimerization and increased lipid rafts in microglia from male mice correlate with the chronic pain phenotype in WT serum injected mice.

## Supplementary Information

Supplementary file 1. 

Supplementary file 2. 

## Data Availability

Data is provided within the manuscript or supplementary information files.

## Abbreviations

ANOVA: analysis of variance
BMD: bone mineral density
BV/TV: trabecular bone volume rate
CGRP: calcitonin gene related peptide
CNS: central nervous system
Ct.Ar: 2D cross sectional bone area
Ct.Th: cortical thickness
CTxB: cholera toxin B
DAPI: 4′,6-diamidino-2-phenylindole
DRG: dorsal root ganglia
GAP43: growth-associated protein-43
GFAP: glial fibrillary acidic protein
IHC: immunohistochemistry
i.p.: intraperitoneal
Iba: ionized calcium binding adaptor molecule 1
OD: optical density
microCT: micro computed tomography
RA: rheumatoid arthritis
ROI: region of interest
Tb.N: trabecular number
TH: tyrosine hydroxylase
TLR: Toll-like receptor
WT: wild type
