# Supplementary figures and images for: Role of TLR4 activation and signaling in bone remodeling, and afferent sprouting in serum transfer arthritis
Source: Arthritis Res Ther. 2024 Dec 18;26:212. doi: 10.1186/s13075-024-03424-4 (PMC11654167; doi:10.1186/s13075-024-03424-4)

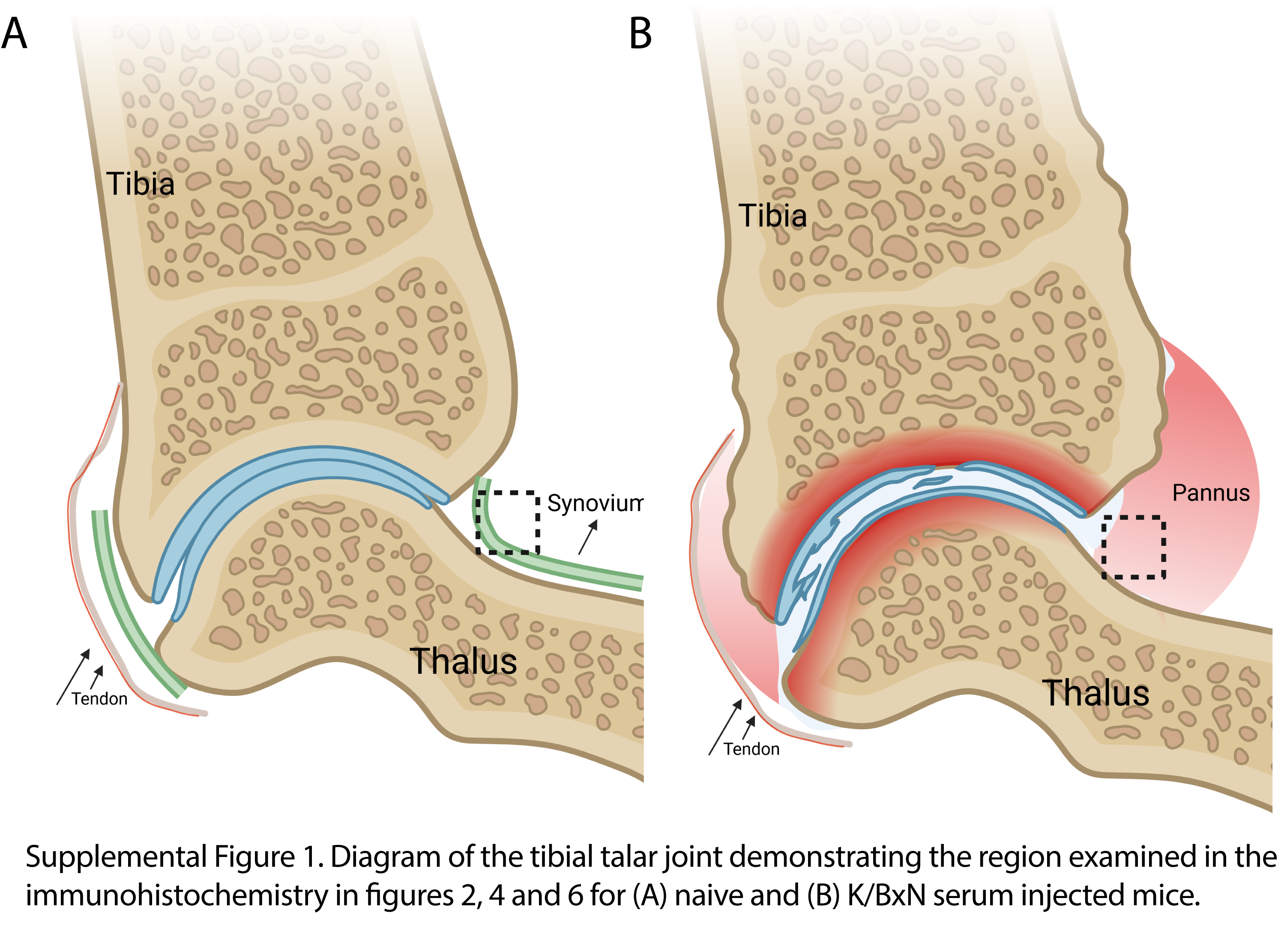

Supplement: Supplementary file 1 [file 13075_2024_3424_MOESM1_ESM.tif]

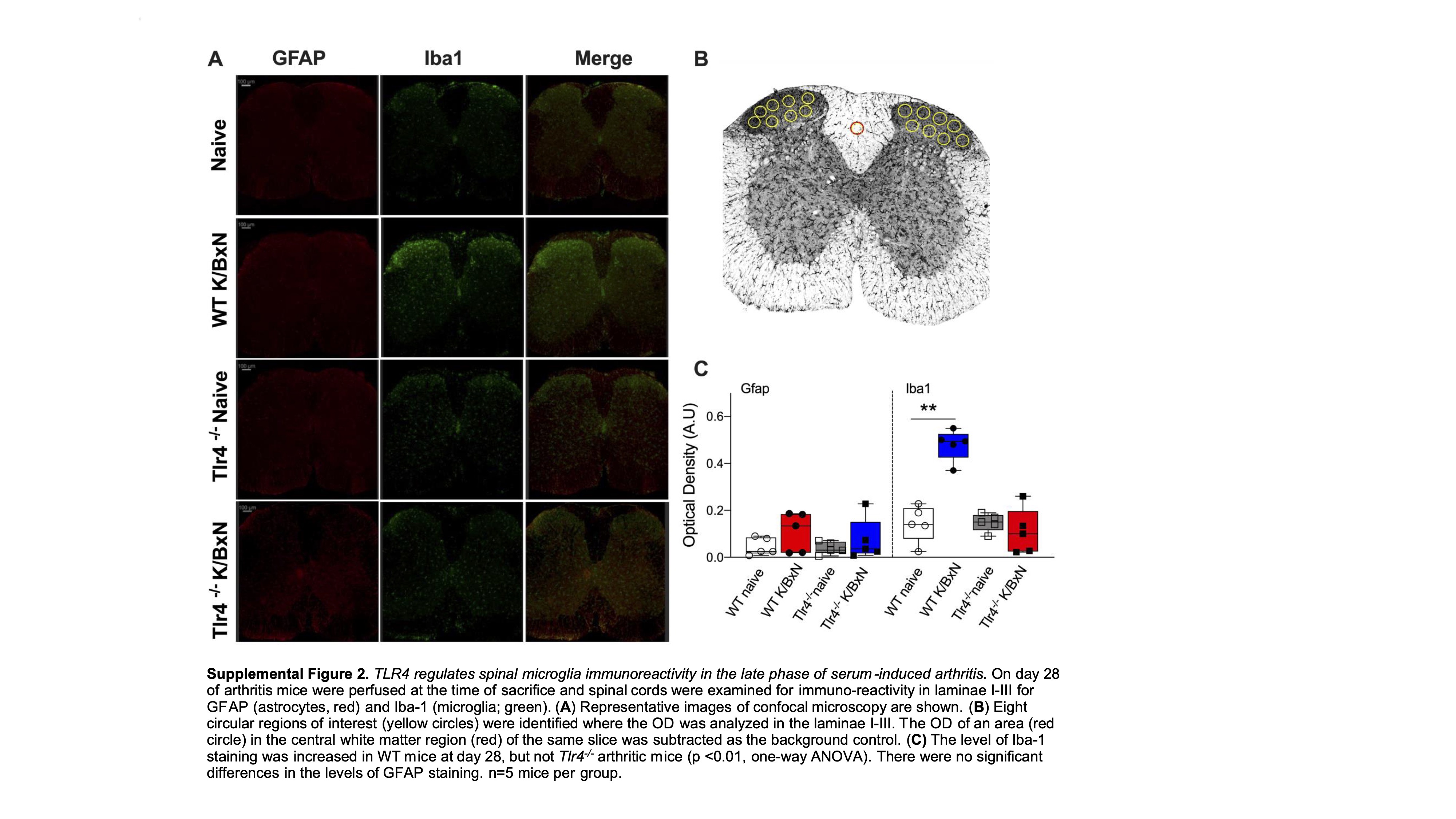

Supplement: Supplementary file 2 [file 13075_2024_3424_MOESM2_ESM.jpg]
